# Contrasting Micro/Nano Architecture on Termite Wings: Two Divergent Strategies for Optimising Success of Colonisation Flights

**DOI:** 10.1371/journal.pone.0024368

**Published:** 2011-09-14

**Authors:** Gregory S. Watson, Bronwen W. Cribb, Jolanta A. Watson

**Affiliations:** 1 School of Pharmacy and Molecular Sciences, James Cook University, Townsville, Australia; 2 Centre for Microscopy and Microanalysis and School of Biological Sciences, University of Queensland, St. Lucia, Australia; RMIT University, Australia

## Abstract

Many termite species typically fly during or shortly after rain periods. Local precipitation will ensure water will be present when establishing a new colony after the initial flight. Here we show how different species of termite utilise two distinct and contrasting strategies for optimising the success of the colonisation flight. *Nasutitermes* sp. and *Microcerotermes* sp. fly during rain periods and adopt hydrophobic structuring/‘technologies’ on their wings to contend with a moving canvas of droplets in daylight hours. *Schedorhinotermes* sp. fly after rain periods (typically at night) and thus do not come into contact with mobile droplets. These termites, in contrast, display hydrophilic structuring on their wings with a small scale roughness which is not dimensionally sufficient to introduce an increase in hydrophobicity. The lack of hydrophobicity allows the termite to be hydrophilicly captured at locations where water may be present in large quantities; sufficient for the initial colonization period. The high wettability of the termite cuticle (*Schedorhinotermes* sp.) indicates that the membrane has a high surface energy and thus will also have strong attractions with solid particles. To investigate this the termite wings were also interacted with both artificial and natural contaminants in the form of hydrophilic silicon beads of various sizes, 4 µm C_18_ beads and three differently structured pollens. These were compared to the superhydrophobic surface of the planthopper (*Desudaba psittacus*) and a native Si wafer surface. The termite cuticle demonstrated higher adhesive interactions with all particles in comparison to those measured on the plant hopper.

## Introduction

Insects demonstrate a remarkable diversity in the way they contend with the elements of nature. For many insects the environmental conditions are harsh and their ability to maintain adequate mobility is vital for survival. How insects interact with water bodies of various sizes is an important aspect as it is seldom possible to escape contact. The water contact angles of insect surfaces show a wide range of variation which is broadly correlated with surface roughness and with habitat [1]–[5].

In the majority of insects such adaptations that occur are structural rather than chemical. As noted even in early studies, it appears ‘easier’ for a species to become adapted to an environment by changing the cuticle surface contours/shape rather than the composition of their surface [6]. There are obvious reasons for this evolutionary route, for example many insects already have a hydrophobic chemistry where contact angles are near the upper limit for smooth surfaces [5], [7], [8].

As might be expected, one of the clearest examples of genuine adaptation of wetting properties to a particular mode of life can be seen in surface-living aquatic species. For example the water strider has nano-structured hairs allowing them to walk on water [7]–[9]. Insects which have a life history which brings them into close contact with water or wetted surfaces may also demonstrate structural adaptations to contend with the environment. For example the cranefly has nano-structured wing and leg hairs and damselflies have many thousands of small stalk-like protuberances on the wing membrane which introduces a roughness to the surface [4], [10]. Some terrestrial insects, and indeed many aquatic insects, have hydrophilic cuticle surfaces. In the case of aquatic insects the need to maintain mobility in water necessitates a hydrophilic cuticle. Some insects even exhibit a patterning of hydrophilic and hydrophobic surface structuring to address a specific function. The desert beetle is a notable example where the surface is used to capture water from the atmosphere [11].

Thus, there is an evolutionary pay-off for such insects to adopt either hydrophobic or hydrophilic ‘technologies’, especially on large surface areas such as the wings. Indeed, insects with a high ratio of wing surface area-to-body mass (SA/M) or which have a close relationship with water generally have water resistant wings as they are more susceptible to the detrimental adhesional contacts. In the worst case scenario the insect can become a victim of permanent immobilization on water or wetted surfaces with a reduced capacity to evade or fight off predators.

**Figure 1:**
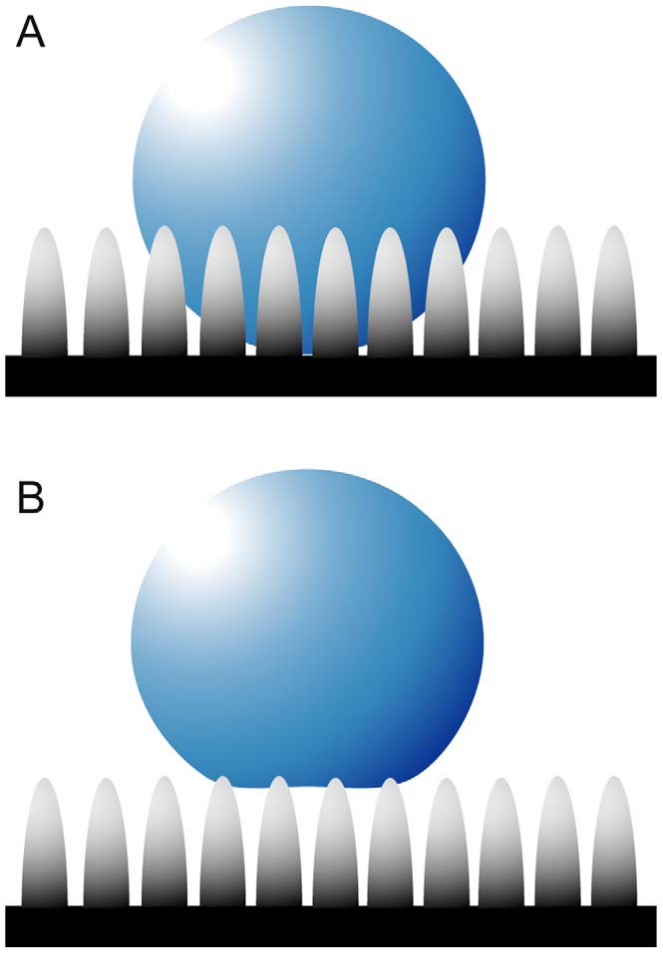
Wenzel and Cassie-Baxter models of surface droplet formation. The diagram shows the interaction of bulk water with a structured surface, according to the (**A**) Wenzel (14) and (**B**) Cassie-Baxter (12) models.

Several theories can be adopted in order to express the wetting interactions on insect cuticle, all of which have certain assumptions and limitations [12]–[16]. The theory by Wenzel [14] makes the assumption that when a liquid drop is placed on a surface consisting of protrusions, the liquid will fill the open spaces as shown in Fig. 1A. This model predicts that roughness of the surface reinforces both hydrophobicity and hydrophilicity. Cassie and Baxter [12], on the other hand, consider the microstructures to be a heterogeneous surface composed of solid and air. The crucial assumption is that the asperities will remain filled with air, thereby allowing the drop to sit on top of the surface as shown in Fig. 1B.

Termites have a simple morphology indicating that they diverged early in insect evolution from a generalized ancestor, undergoing gradual metamorphosis, and acquired the ability to fold the wings flat over the back. Although simple in morphology they are advanced in social behaviour. The alates (winged termites) develop from nymphs by growing wings and compound eyes. The alates of each species fly at a unique time of day, under specific conditions, and shed their wings voluntarily along a basal suture, after which mating and nest settlement commences [17], [18].

Termites have an extremely high SA/M value in relation to many other insect species and typically fly from the nest during rain periods [19]. These two features, together with the fact that termites are typically very weak fliers, indicate that the insect would or should adopt specialised hydrophobic structures on their wings. This would presumably optimise the chances of the colonisation flight which, even though it is generally of a short duration and distance, is critical in the establishment of new colonies [20]. Flying during rain periods may have certain advantages for the insect such as decreasing the likelihood of predator attack due to a mobile canvas of moving droplets disguising individuals on wing or inclement weather reducing predator numbers. As well, local precipitation will ensure water will be present when establishing a new colony after the initial flight. Water is essential for building nests and soil tunnels and nest-founding reproductive termites that use soil look for moist soil in which to burrow [18]. Termite alates typically have large quantities of stored nutrients but reduce weight by flying with minimal water content and rehydrate during the initial stages of colony foundation [17], [20].

This paper demonstrates distinct differences in the termite cuticle (nano)structuring revealing the wetting properties (both hydrophilic and hydrophobic) of different termite species which make their maiden flights under different environmental conditions. We have demonstrated specific interactions of water with the three termite species. The two hydrophobic species (*Nasutitermes* sp. and *Microcerotermes* sp.) exhibit a hierarchical arrangement of the cuticle optimized as an anti wetting surface. A hair array combined with a star-shaped micraster array demonstrates an elegant hierarchical designed approach for minimising interaction with water bodies at various length scales. The dynamic changes occurring from micro-droplet evaporation shows a transitional change from the Cassie-Baxter to Wenzel states which may be promoted by meniscus bridging. The hydrophilic termite species (*Schedorhinotermes* sp.) showed a small scale roughness which was not dimensionally sufficient (in height and/or spacing) to introduce an increase in hydrophobicity. Thus the surface is most likely in the fully wetted Wenzel state. Adhesion with solid anthropogenic and natural particles confirms that the cuticle represents a high surface energy membrane.

The two radically different strategies incorporated by the hydrophobic and hydrophilic termites highlighted by the completely different topographies and wetting behavior is reflected in the insect behavioral responses during the colonisational flight. The hydrophobic species fly during daylight hours under the camouflage of moving droplets while the hydrophilic species flies under the cover of darkness and in the absence of droplets but shortly after rain has occurred. Both strategies tend to increase the chances of sufficient water being available after the colonisational flight and provide us with a better understanding of the way naturally occurring micro/nano structures are used in biology.

## Results and Discussion

**Figure 2:**
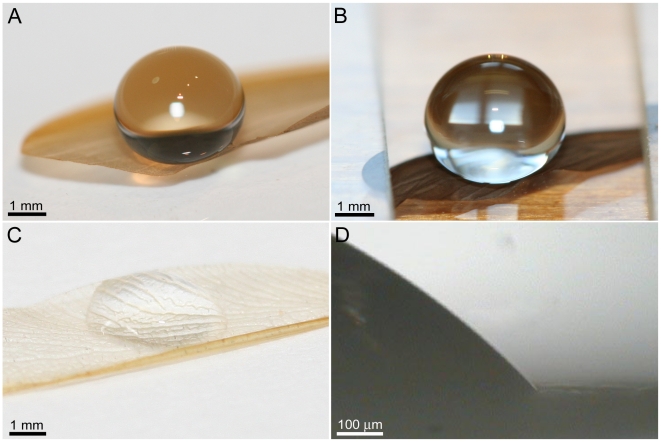
Droplet interaction with 3 different termite wing surfaces. Optical photographs of 10 µL water droplets on the wing membranes of: (**A**) *Nasutitermes* sp. (**B**) *Microcerotermes* sp. and, (**C**) *Schedorhinotermes* sp. (**D**) A higher resolution, side view, optical microscope image revealing the hydrophilic nature of the surface membrane of *Schedorhinotermes* sp.

Optical images demonstrating the interaction of small droplets of water with the wing membrane of the three different termites studied (*Nasutitermes* sp., *Microcerotermes* sp. and *Schedorhinotermes* sp.) are shown in Fig. 2A, B and C, respectively. The droplets on *Nasutitermes sp. and Microcerotermes* sp exhibit remarkable apparent contact angles (CA) of 180° with the underlying membrane. In stark contrast *Schedorhinotermes sp.* has a hydrophilic wing membrane with a contact angle of 70–82° (Fig. 2D).

**Figure 3:**
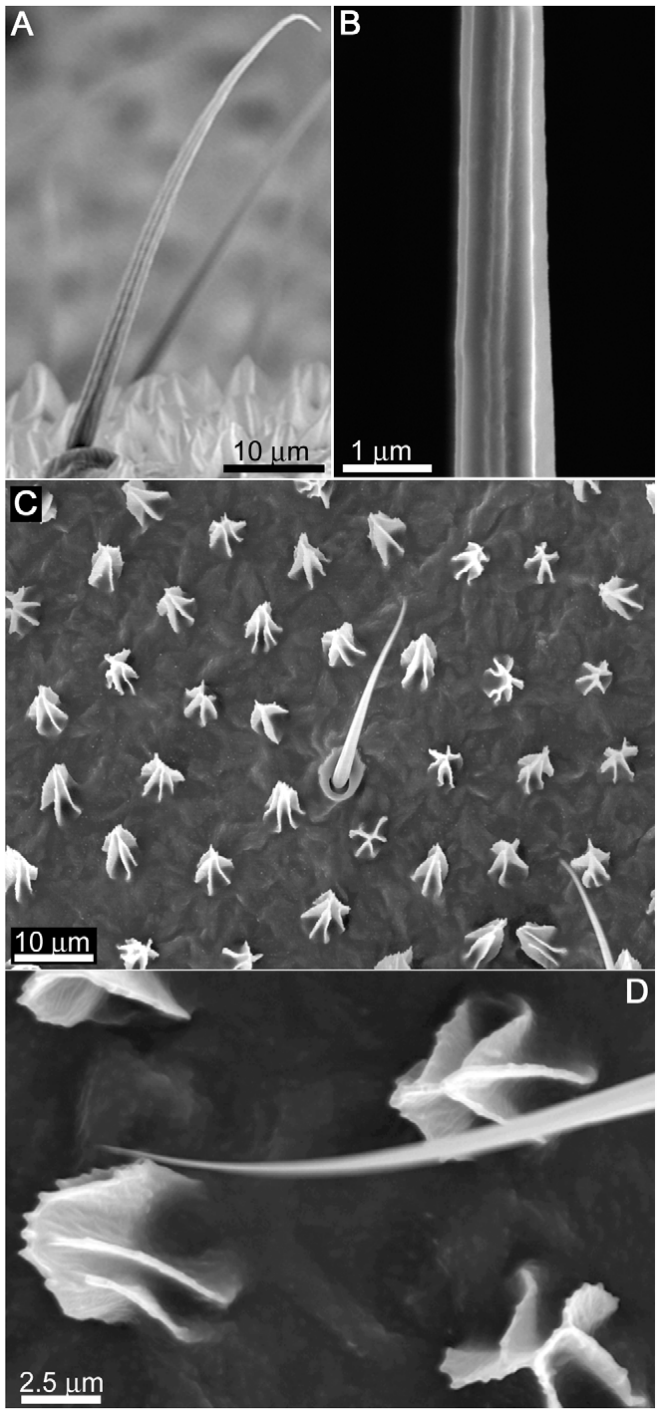
SEM images of the surface structuring on the *Nasutitermes* sp. termite wing membrane. (**A**) Side view image revealing hairs (macrotrichia) in sockets protruding from the surface. (**B**) High resolution image of the macrotrichia revealing a series of nano grooves aligned along the long axis on the hairs. (**C**) A topographical view of the micrasters and macrotrichia on the wing membrane, both exhibiting a sheet-like structuring, with (**D**) revealing the finer nano-structuring on the surface of the micrasters.

The spherical droplets shown in Fig. 2A and B on the surfaces of the wing cuticle of *Nasutitermes* sp. and *Microcerotermes* are generally not stable and upon contact will move laterally across the surface. Mobile droplets will bounce off the membrane on contact. The droplets are supported by many membrane hairs (macrotrichia) extending from the wing membrane as shown in Fig. 3A. We have previously examined the surfaces of these two termites and shown that the ability of these hairs to hold droplets above the cuticle is enhanced by nano-structuring of the hairs as seen in Fig. 3B [19]. The density of hairs on the termite wing can be as high as 5 per 100 µm^2^ yielding many thousands of hairs per single wing surface. As little as 100 hairs can easily support the weight of a 10 µl droplet for minor hair deflections of less than 10 µm [19].

Even though the hairs serve as an antiwetting array, they may serve additional functions for the termite. Hairs on various insects have been shown to serve multi-functional purposes such as protection against wetting, minimising contact with solid surfaces and in some cases have been attributed to aerodynamic factors [21]–[23]. Interestingly a smaller scale structuring also exists on the wing membrane (between the hairs on the termite wing, as seen in Fig. 3A, C and D). These structures, termed micrasters, have been examined on a number of termite species [24]–[26]. The micrasters in Fig. 3 exhibit a skeletal framework comprised of 5–7 distinct arms consisting of uniformly thin sheets around 90–120 nm in width, many of which originate from the same central location on the star structure and have a secondary nano-roughness on the top ridges (Fig. 3D). They are typically 5–6 µm in height at the highest point and have a width (extremity of arm to arm distance) generally of 5–6 µm. The centre point-centre point spacing of structures is ∼10 µm (see Table 1).

**Table 1:** Geometrical parameters of insect species investigated in this study.

Insect (*Species*)	Structure Type	Height	Density (µm^−2^)	Max Width	Spacing/periodicity
**Hydrophilic termite** (*Schedorhinotermes* sp.)	Domes	150±5 nm	0.01	295±10 nm	850±25 nm
**Hydrophobic termite** (*Nasutitermes* sp. and *Microcerotermes* sp.)	Macrotrichia	70±5 µm	0.00015	2.4–2.6 µm	100±50 µm
	Micraster	5.5±0.5 µm	0.008	5–6 µm	9.5±1 µm
**Planthopper** (*Desudaba psittacus*)	Rods	410±70 nm	52	60±10 nm	145±10 nm

**Figure 4:**
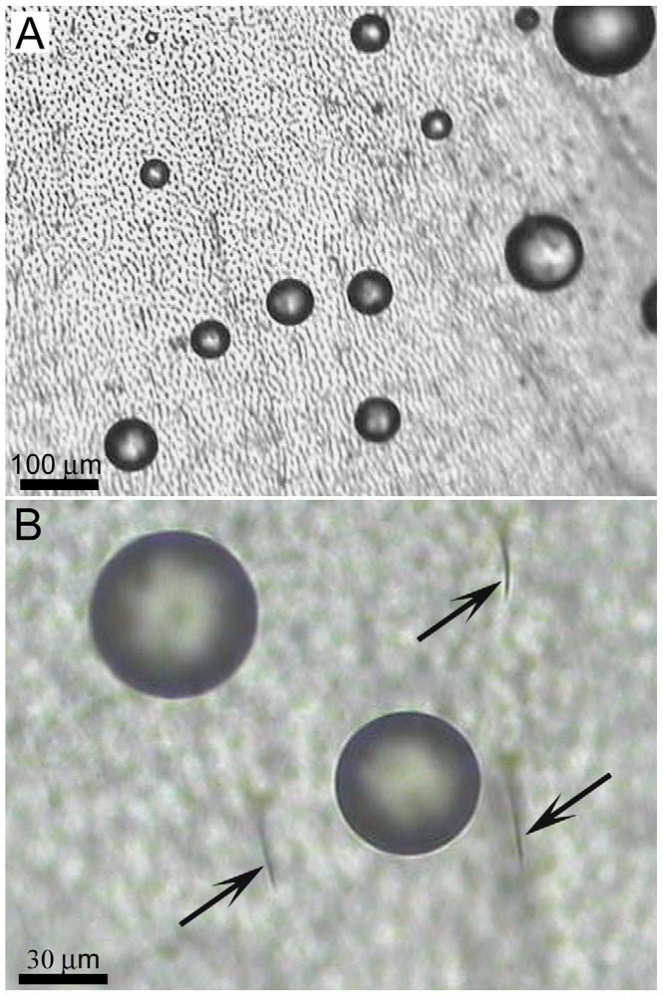
Optical microscope images showing micro-droplets resting on the termite wing *Microcerotermes* sp. The droplets maintain their spherical shape and occupy regions between the hair arrays (which are highlighted in (**B**) by the arrows).

**Figure 5:**
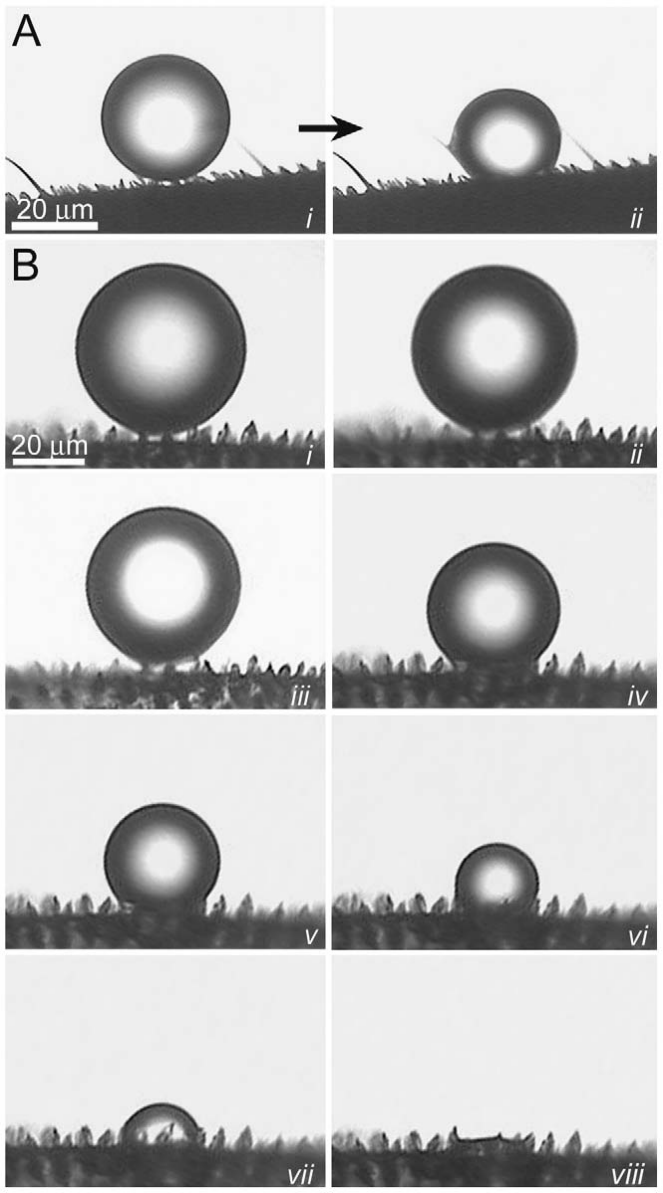
Micro droplet progression from the Cassie-Baxter to Wenzel states of interaction. (**A**) shows the interaction of a small micro-sized water droplet evaporating on the termite wing membrane of *Microcerotermes* sp. A detailed progression of droplet evaporation as it makes a transition between the Cassie-Baxter to the Wenzel state is shown in (**B**), from parts *i* to *viii*.

**Figure 6:**
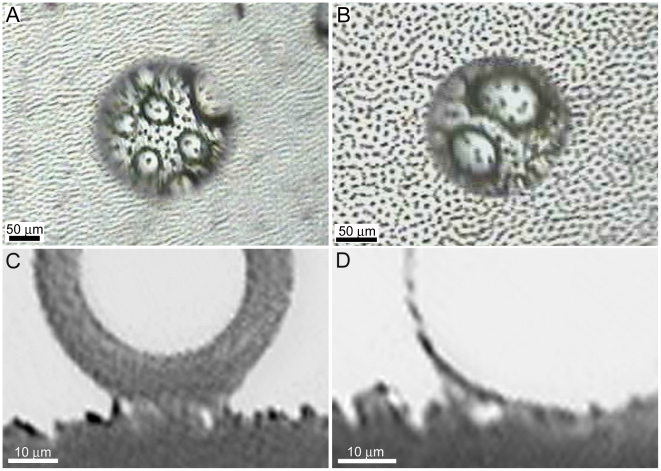
Images revealing meniscus bridging between micro droplets and the *Microcerotermes* sp. micrasters. (**A**) and (**B**) Top views (through the droplets), and (**C**) and (**D**) side views of droplets showing meniscus bridging on the surface of the termite membrane during the Cassie-Baxter and Wenzel transitional states.

The micrasters can support droplets of water which are small enough to fit between the hair arrays and can potentially make contact with the underlying wing membrane (Fig. 4A and B). We have previously reported on the micro-droplets at the initial contact with the termite membrane [19] however no temporal studies have been reported. In order to investigate the dynamic interaction of micrasters over time with water, micro-droplets (20–150 µm in diameter) were sprayed onto the wing surfaces. As micro droplets are sprayed onto the surface of the termite wing, the droplets form a Cassie-Baxter state of interaction as seen in Fig. 5A and B (parts *i*). If, however, droplets are left to evaporate, then a transition occurs from the Cassie-Baxter to Wenzel state (Fig. 5A (parts *i* to *ii*), B (parts *i* to *viii*) and movie S1). A recent study has examined droplet transitions from the Cassie to Wenzel state on fabricated silicon structures coated with PF_3_ with dimensions not too dissimilar to the termite structuring [27], [28]. The fabricated structures had a width and spacing of 5 µm diameter and 7 µm, respectively, and droplets evaporating made the transition at around a diameter of 40 µm. This is similar to the transition size of droplets on the termite structures (Fig. 5A and B), however the fabricated structures were around twice the height (10 µm height) of the termite micrasters. The study showed that the higher the structure height the easier it is to maintain the Cassie state. Thus even though the termite micro-structures are significantly shorter in height, and importantly not of a solid construction, they are still comparable to the fabricated structures. Optical microscopy indicates that transition from the Cassie to the Wenzel orientation may be facilitated from unstable meniscus bridging. Fig. 6A–D shows meniscus bridging prior to the transition process. Optical microscopy showed that micro droplets that were in the Cassie-Baxter state were removed from the membrane surface by minor vibrations/movements of the wings facilitated by minimal adhesion with the micrasters. As well, larger droplets resting on the hairs absorb micro-droplets resting on the micrasters (Fig. S1).

**Figure 7:**
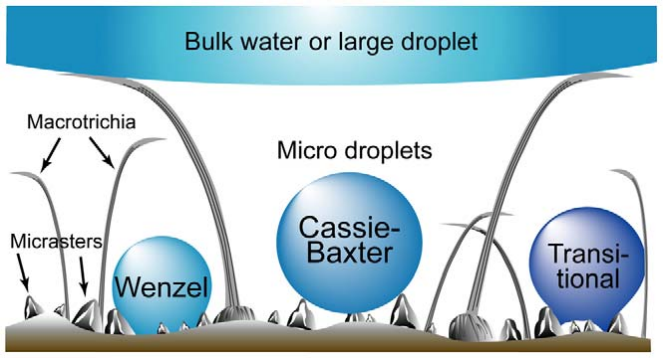
Anti-wetting hierarchical arrangement of the hair/micrasters demonstrating various wetting states.

Fig. 7 shows diagrammatically the anti-wetting arrangement on the termite wing with the various wetting states on the micrasters. The specialised topographies are designed for minimising the solid-liquid contact area and maximising the liquid-air contact. The hair/micraster array demonstrates an elegant hierarchical designed approach for minimising interaction with water bodies of various length scales.

As well, the open membrane hierarchy demonstrates a design for achieving this state utilising minimal structural material and thus reduced weight for the insect. All the superhydrophobic termites used in this study were collected during flight in the rain in daylight hours (5 separate occasions) e.g., see movie S2. This indicates that the termites may use the mobile canvas as a camouflage backdrop for the colonisation flight. It also demonstrates that the insects can easily cope with rain where flight has to be maintained. As termites are not typically good fliers and have a low wing flapping rate, the ‘shedding efficiency’ of water on the surface, and thus interaction time with droplets may be critical to maintaining controlled flight. Termites which land or drop to a wetted surface (e.g., droplet forces propelling the termite off its flight path) have been observed to escape even though their wing came into contact with the wetted surface during escape (see movie S3).

**Figure 8:**
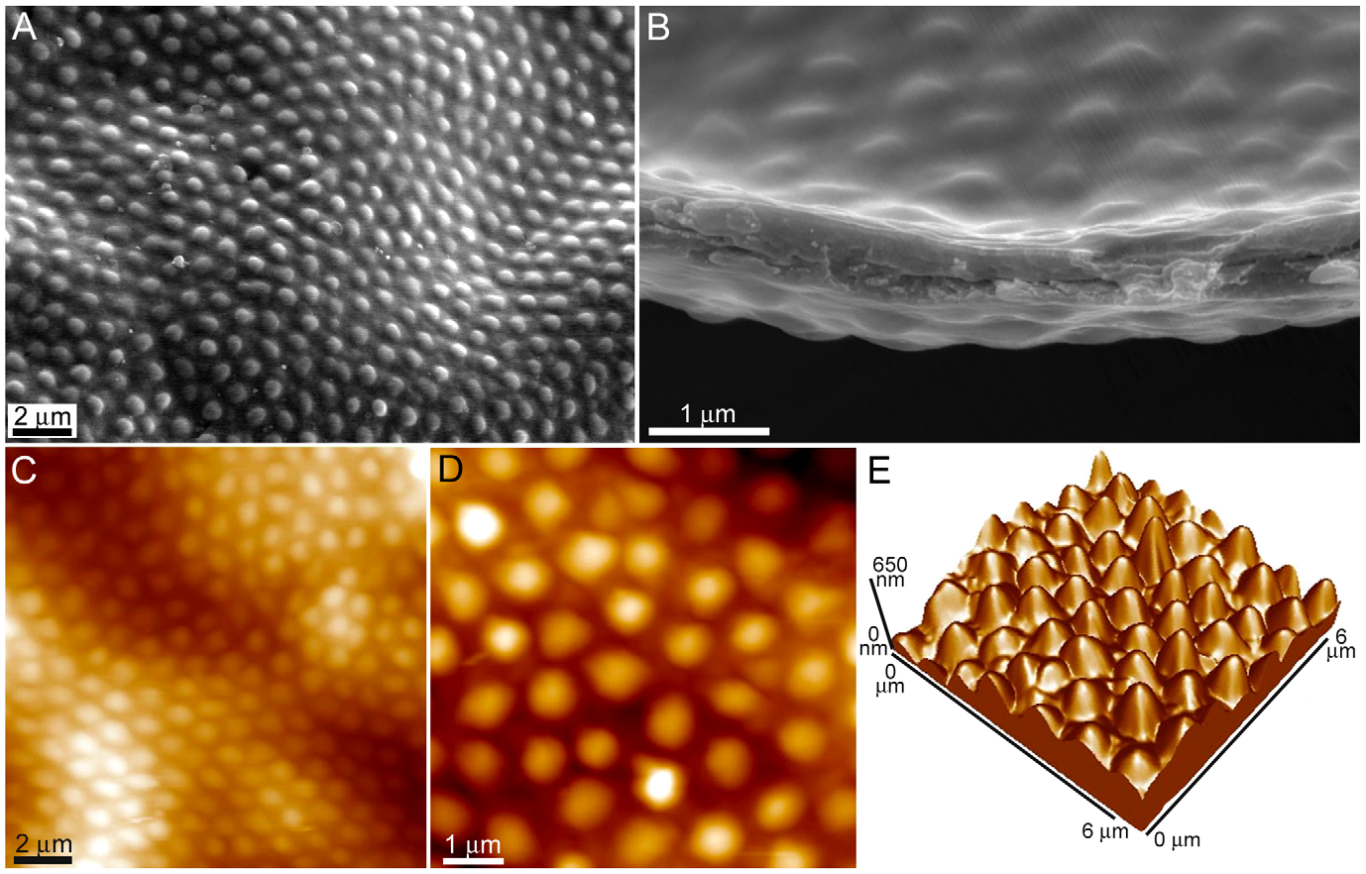
Topographical images of the surface structure of the hydrophilic *Schedorhinotermes* sp. termite wing. (**A**) A topographical and (**B**) cross-sectional SEM image of the wing surface of the termite *Schedorhinotermes* sp. not only revealing the hexagonal close packed arrangement of the protuberances, but also the folds and ridges of the wing membrane. (**C**) The two topographical AFM images in parts (**D**) and (**E**) and 3-dimentional image in (**F**) reveal the protuberance height and spacing in greater detail.

The topography of the contrasting wing membrane of the hydrophilic termite in this study (*Schedorhinotermes* sp.) is shown in Fig. 8. The surface reveals topography in a hexagonal array arrangement with micron sized curved projections (protuberances) spaced 700–1000 nm apart (centre to centre distance) and 150–250 nm in height (Fig. 8E). It is also evident that the wing structuring consists of folds and ridges. Kesel [29] has shown that folds and ridges in the case of a dragonfly wing provide more stability and have an advantageous aerodynamic effect. The termite wing membrane is quite thin (see the cross-sectional image in Fig. 8B) in relation to many other insects (less than 1 µm compared to 5–10 µm for a typical cicada). Thus the surface on the termite wing may serve a similar function of aiding stability and flying efficiency. A previous study has suggested that the trough regions on the termite wing may assist in capturing air and help prevent its premature separation while the array of ‘bumps’ may act as a series of stabilizing elements designed to handle loading forces. Even though the membrane is quite thin, the membrane retains a higher than expected rigidity [30]. Future studies in relation to stiffness and aerodynamics are required in order to ascertain the influence from these structural components.

*Schedorhinotermes* sp. was collected on 14 separate occasions and was never observed to fly in the rain or during daylight hours. This suggests the termite may use the cover of darkness to minimise predation instead of moving droplets as camouflage as utilised by the superhydrophobic termite species. On all of these occasions precipitation was observed usually within a 72 hour period prior to flights.

**Figure 9:**
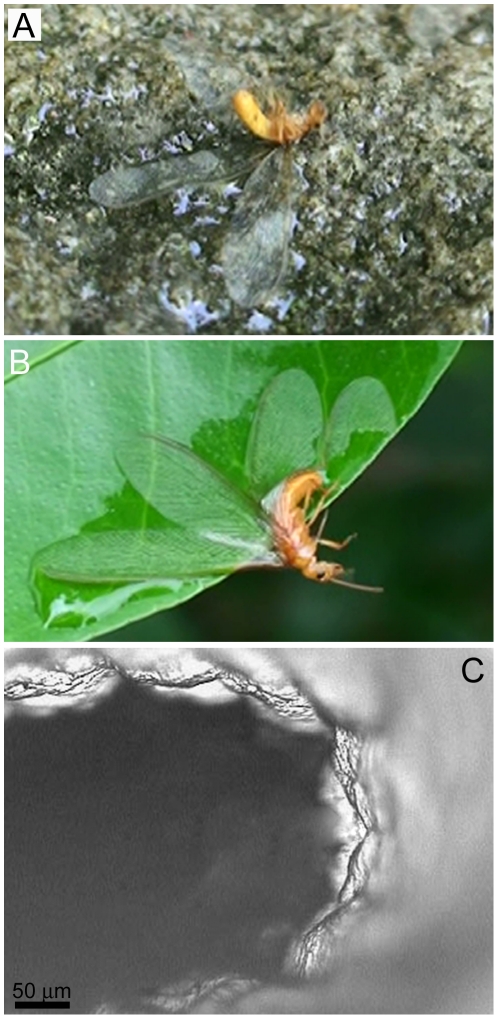
Hydrophilic termite immobilisation and SEM image of membrane folding. The hydrophilic termite gets pinned to various wetted surfaces, e.g., rock (**A**) or leaf (**B**). The wing membrane also tends to fold as seen in the higher resolution SEM image in (**C**).

We have observed many hundreds of these termites (427 on one single occasion, over an area of 20 m^2^) which have become immobilised by bulk water (small ponds/puddles) or by capillary action on foliage or substrate (for example see Fig. 9A and B). The insect is typically unable to free themself from the adhesional forces (see also movie S4 and S5). Wetting of the wings can also induce membrane folding which also effectively impedes insect flight (Fig. 9C). But such capture has the advantage of terminating flight at a location which may provide sufficient water for successfully establishing a new nest.

These observations indicate that the termite uses a strategy which adopts *hydrophilic* instead of hydrophobic mechanisms for maximising success of the colonisation flight. It is apparent from the wetting behaviour that the small scale roughness on the *Schedorhinotermes* sp. is not dimensionally sufficient (in height and/or spacing) to introduce an increase in hydrophobicity. High resolution optical microscopy of the termite surface membrane revealed that the surface is fully wetted in the Wenzel state (i.e. no air layer could be observed).

**Figure 10:**
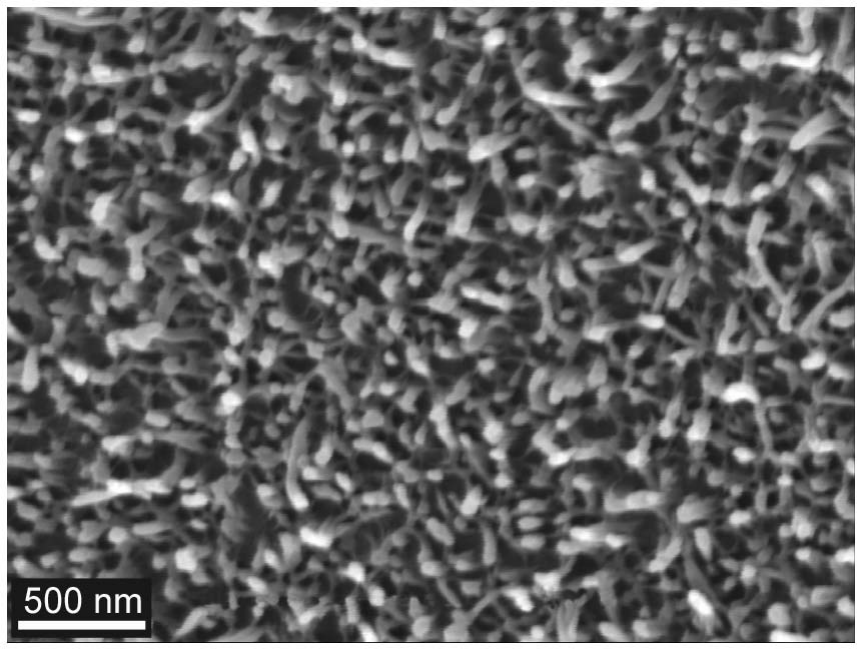
SEM image of the fine rod-like nano-structuring on the wing of the planthopper *Desudaba psittacus*.

The high wettability of the termite cuticle (*Schedorhinotermes* sp.) suggests that the membrane has a high surface energy and will also show a strong adhesion with solid particles. To investigate this further we studied the adhesional properties with natural and artificial particles of various sizes. For comparison we have also measured the adhesion on the wing of a planthopper (*Desudaba psittacus*). The planthopper was chosen for comparison due to its anti-wetting/superhydrophobic properties (CA 156°) and membrane topography. Comparison of adhesion with the other two superhydrophobic termites in this study is difficult due to the hierarchical nature of the membrane landscape (i.e. it is difficult to isolate the contributions of micrasters from the hairs). In contrast the planthopper (*Desudaba psittacus*) revealed a less hierarchically-structured surface topography with rod-like structures forming a layer of porous matting consisting of rods less than 100 nm in diameter as shown in Fig. 10. These structures are similar to those found on damselflies [6]. In that study the authors suggested a number of possible functions for the ‘wax-like’ covering including intra/inter-specific communication based on ultraviolet light reflection of the layer. The covering was also suggested to protect against the insect when in contact with water.

Four different sized particles (three with hydrophilic chemistries) have been used to measure adhesion on the insect wing membranes. The dimensional and chemical differences were chosen to mimic contact conditions of particles which could potentially contaminate the structured insect cuticle surfaces. As well, natural particles (3 different pollens) were interacted on the surfaces. The pollens (*Pimelea linifolia ssp*, *Grevillea Red Sunset* and *Acacia fimbriata* shown in Fig. S2A–C, respectively) were chosen based on the 3 distinct topographies with various levels of roughness.

**Figure 11:**
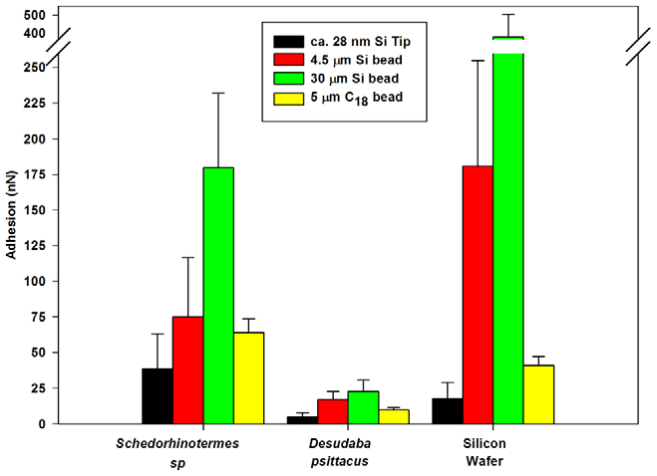
Adhesive values between various beads interacting with the termite, planthopper and Si surfaces. The interactions were between the hydrophilic termite (*Schedorhinotermes* sp.) and planthopper (*Desudaba psittacus*) wing membranes, and a silicon wafer surface and 5 µm C_18_, 30 µm and 5 µm beads, and ca. 28 nm AFM tip.

**Figure 12:**
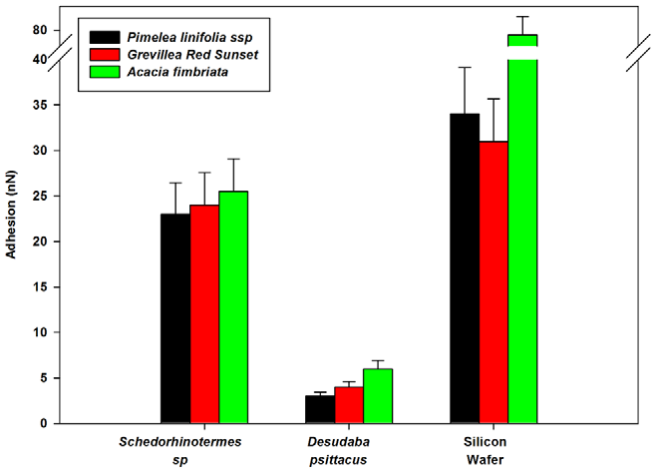
Adhesive values between three different pollen grains interacting with the termite, planthopper and Si surfaces. The pollens include *Pimelea linifolia* ssp., *Grevillea Red Sunset* and *Acacia fimbriata*.

The adhesion on the planthopper was significantly less than on the termite for all sizes and chemistry of particles (Fig. 11 & 12). The adhesion between the silica tip/microsphere and the insect cuticles represents a high surface energy contaminant particle coming into contact with low energy hydrophobic (planthopper) and higher energy hydrophilic micro/nano-structuring (termite). The larger the particle contacts (e.g., compare Silica AFM tip nm's, 4.5 and 30 µm sphere – Fig. 11) the higher the adhesion which reflects the increase in radius of curvature and increased contact points. Thus the real contact area increases along with the meniscus contributions. This is highlighted in Fig. 11 with the differences in adhesion and wetting between the two insects. Particle adhesion on the superhydrophobic plant hopper cuticle was also much lower in comparison with that for a topographically flat hydrophilic Si surface. The higher adhesion values measured between the contacting surfaces of the termite cuticle/silicon surface and the hydrophilic contaminants reflects the menisci formation from liquid present on the surfaces. As well, the relatively flattened and broadened structures of the termite membrane does not minimise the contact area to the degree of the planthopper species where solid-solid contacts are significantly reduced by the thin rod-like structuring. Adhesion of the C_18_ particles with the insect cuticles was less than the comparably sized Silica particles (compare data from Fig. 11). This represents the difference in adhesion of a hydrophobic and hydrophilic particle coming into contact with the insect wing membranes.

The pollen particles investigated were of a similar size scale to the 30 µm silicon beads. However due to the rougher morphology and more hydrophobic nature of the long chain polymers that composes the pollen sporopollenin (outer layer) adhesion between the pollens and the insect cuticles is lower (see Fig. 12). The spherical shaped profile with small micro asperities *Pimelea linifolia* ssp exhibited the lowest adhesion reflecting a reduced contact area between the surfaces. Interestingly, the adhesion between termite wing and the pollens was of a similar order to adhesion measurements of pollens to stigma cells (in the order on 10^−7^ N) [31]. The high adhesion values measured on the termite suggests a higher risk of wing contamination although this may be of limited concern as the insect will only utilise the wings for relatively short periods before completely shedding them.

## Materials and Methods

### Ethics Statement

No specific permits were required for the described field studies. The insect and plant species collected are not endangered or protected.

### Sample collection

Insect and plant samples were collected on the private residences of Drs. GS and JA Watson (authors) in Brisbane, QLD Australia, and Townsville, QLD, Australia.

### Photographic and video imaging

Photographs of droplets resting on single excised wings were obtained using a Canon Digital 350D SLR, and Canon Ultrasonic EF-S 60 mm macro lens at an 8 megapixel resolution. The photographs were cropped with no further image processing and scale bars were applied using Photoshop. Videos were captured using a Sony HD video recorder (HDR-CX7) in AVCHD format (MPEG-4 AVC/H.264) and converted from MPEG-2 to .mpg format using Quick Media Converter, v3.6.0. The original video resolution was 1440×1080.

### Optical microscopy

Imaging on single excised termite and planthopper wings were obtained using an AIS – Optical Microscope VG8 coupled with a Panasonic colour CCTV camera WV-CP410/G attached allowing image capturing. Top views were captured with the microscope placed in a vertical position, with side views obtained with the microscope in a horizontal position with a 40× magnification.

### Scanning Electron Microscopy

In the case of scanning electron microscope (SEM) imaging, wing tissues of the insects (approx. 3 mm×3 mm) were excised and placed on an aluminium pin-type stub with carbon-impregnated double-sided adhesive, then sputter coated with 7–10 nm of platinum, before being imaged using a JEOL 6300 field emission SEM at 8 kV.

### Atomic Force Microscopy

Topographical images were obtained using a JEOL JSPM-4200, at a constant force in contact mode with a lever-imposed normal force in the range 5–15 nN. The scanning rate in the fast-scan direction was ca. 3 Hz, and a typical image was composed of 500×500 pixels. A TopoMetrix (Veeco Instruments) Explorer TMX-2000 AFM was used to obtain the adhesion data. This was carried out in the Force versus distance (F-d) mode. A 130×130 µm^2^ tripod air scanner was used with a *z* range of 9.7 µm. The analyses were carried out under air-ambient conditions (temperature of 20–25°C and 60–75% RH) and thus the strongest contributing force of attraction is from capillary forces from the surfaces [32]. ‘Beam-shaped’ tipless levers (NT-MDT Ultrasharp) were used for the attachment of beads and pollens. The force constants of levers (*k_N_*) were determined by accepted methods [33]. The scanners were calibrated using a known standard [34].

Force versus distance (f-d) analysis requires the tip/particle to be held stationary at an x–y (sample plane) location and ramped along the *z*-axis, first in the direction of approach and contact with the surface, and then in the reverse direction. F-d curves were acquired at rates of translation in the z-direction in the range 2–5 µm s^−1^. Each f-d curve consisted of 600 data points. The attachment procedure for particle adhesion has been described in the literature [35]. Twenty five measurements per particle (micro particle or nano tip)-substrate size combination were acquired. A total of 5 particles were attached to cantilevers for each particle type (e.g., five silica beads of ∼4.5 µm diameter were used for adhesion measurements each yielding 25 measurements). Only pollen grains which exhibited the same orientation upon fixing to a lever were used for adhesion measurements.

## Supporting Information

Figure S1 **Optical microscope image showing large sized droplets resting on the wing surface of** ***Nasutitermes*** **sp.**
(TIF)

Figure S2 **SEM images revealing the surface topographies of the three pollen grains** ((**A**) *Pimelea linifolia* ssp. (**B**) *Grevillea Red Sunset* (**C**) *Acacia fimbriata*) attached to AFM tipless beam shaped levers.
(TIF)

Movie S1 **Droplet transition from the Cassie-Baxter to Wenzel states of interactions** between a micro-droplet and micrasters found on the wing of *Microcerotermes* sp.
(MPG)

Movie S2 **Colonisation flight of the hydrophobic termite** ***Microcerotermes*** **sp.** under a canvas of rain, avoiding predator attack.
(MPG)

Movie S3 **The escape of the superhydrophobic termite (** ***Microcerotermes*** **sp.) off the surface of a small puddle.**
(MPG)

Movie S4 **Result of the colonisation flight of the hydrophilic termite** ***Schedorhinotermes*** **sp.** The insect is pinned to the surface of a wetted rock.
(MPG)

Movie S5 **Result of the colonisation flight of the hydrophilic termite** ***Schedorhinotermes*** **sp.** The insect is pinned to the surface of a wetted citrus leaf.
(MPG)

## References

[1] Sun T, Feng L, Gao X, Jiang L (2005). Bioinspired surfaces with special wettability.. Acc Chem Res.

[2] Wagner P, Neinhuis C, Barthlott W (1996). Wettability and contaminability of insect wings as a function of their surface sculptures.. Acta Zoologica.

[3] Cong Q, Chen G-H, Fang Y, Ren L-Q (2004). Study on the super-hydrophobic characteristic of butterfly wing surface.. J Bionics Eng.

[4] Gorb SN, Kesel A, Berger J (2000). Microsculpture of the wing surface in Odonata: Evidence for cuticular wax covering.. Arthropod Structure & Development.

[5] Watson GS, Myhra S, Cribb BW, Watson JA (2008). Putative function(s) and functional efficiency of ordered cuticular nano-arrays on insect wings.. Biophys J.

[6] Holdgate MW (1955). The wetting of insect cuticles by water.. J Exp Biol.

[7] Gao X, Jiang L (2004). Water-repellent legs of water striders.. Nature.

[8] Feng X-Q, Gao X, Wu Z, Jiang L, Zheng Q-S (2007). Superior water repellency of water strider legs with hierarchical structures: Experiments and analysis.. Langmuir.

[9] Watson GS, Cribb BW, Watson JA (2010). Experimental determination of the efficiency of nanostructuring on non-wetting legs of the water strider.. Acta Biomaterialia.

[10] Hu H-MS, Watson GS, Cribb BW, Watson JA (2011). Non wetting wings and legs of the cranefly aided by fine structures of the cuticle.. J Exp Biol.

[11] Parker AR, Lawrence CR (2001). Water capture by a desert beetle.. Nature.

[12] Cassie ABD, Baxter S (1944). Wettability of porous surfaces.. Trans Faraday Soc.

[13] Gao L, McCarthy TJ (2007). How Wenzel and Cassie were wrong.. Langmuir.

[14] Wenzel RN (1936). Resistance of solid surfaces to wetting by water.. Ind Eng Chem.

[15] Herminghaus S (2000). Roughness-induced non-wetting.. Europhys Lett.

[16] Shirtcliffe NJ, McHale G, Newton MI (2010). An introduction to superhydrophobicity.. Adv Colloid Interface Sci.

[17] van Achterberg K, Aspock H, Aspock U, Baderson J, Britton EB (1991). The Insects of Australia: A Textbook for Students and Research Workers.

[18] Pearce MJ (1997). Termites Biology and Pest Management.

[19] Watson GS, Cribb BW, Watson JA (2010). How micro/nanoarchitecture facilitates anti-wetting: An elegant hierarchiacal design on the termite wing.. ACS Nano.

[20] Nalepa CA, Miller LR, Lenz M (2001). Flight characteristics of *Mastotermes darwiniensis* (Isoptera, Mastotermitidae).. Insectes soc.

[21] Gorb S (2001). Attachment Devices of Insect Cuticle.

[22] Marden JH, Kramer MG (1994). Surface-skimming stoneflies: A possible intermediate stage in insect flight evolution.. Science.

[23] Masters WM, Eisner T (1990). The escape strategy of green lacewings from orb webs.. J Insect Behaviour.

[24] Roonwal ML (1985). Wing microsculpturing in termites (Isoptera) under the Scanning Electron Microscope.. Zool Anz Jena.

[25] Rathore NS (1977). Third study of evolution and systematic significance of wing micro-sculpturing in termites. Micrasters in some Thinotermitidae and Termitidae.. Zool Anz.

[26] Rathore NS (1974). On a new systematic character in termites, the microsters.. Z Zool Syst Evolutionsforsch Berlin.

[27] Bhushan B, Jung YC (2011). Natural and biomimetic artificial surfaces for superhydrophobicity self-cleaning, low adhesion, and drag reduction.. Progress in Materials Science.

[28] Bhushan B, Jung YC (2008). Wetting, adhesion and friction of superhydrophobic and hydrophilic leaves and fabricated micro/nanopatterned surfaces.. J Phys: Condens Matter.

[29] Kesel AB (2000). Aerodynamic characteristics of dragonfly wing sections compared with technical aerofoils.. J Exp Biol.

[30] Watson GS, Watson JA (2004). Natural nano-structures on insects - Possible functions of ordered arrays characterized by atomic force microscopy.. App Surf Sci.

[31] Zinkl GM, Zwiebel BI, Grier DG, Preuss D (1999). Pollen-stigma adhesion in *Arabidopsis*: A species-specific interaction mediated by lipophilic molecules in the pollen exine.. Development.

[32] Blach-Watson JA, Watson GS, Brown CL, Myhra S (2004). UV patterning of polyimide: Differentiation and characterization of surface chemistry and structure.. App Surf Sci.

[33] Cleveland JP, Manne S, Bocek D, Hansma PK (1993). A non-destructive method for determining the spring constant of cantilevers for Scanning Force Microscopy.. Rev Sci Instrum.

[34] Watson GS, Dinte BP, Blach JA, Myhra S (2002). Demonstration of atomic scale stick-slip events stimulated by the force versus distance mode using atomic force microscopy.. J Phys D – Appl Phys.

[35] Watson GS, Blach JA, Cahill C, Nicolau DV, Pham DK (2004). Interactions of poly(amino acids) in aqueous solution with charged model surfaces – Analysis by colloidal probe.. Biosensors & Bioelectronics.

