# Supplementary figures and images for: Contrasting Micro/Nano Architecture on Termite Wings: Two Divergent Strategies for Optimising Success of Colonisation Flights
Source: PLoS One. 2011 Sep 14;6(9):e24368. doi: 10.1371/journal.pone.0024368 (PMC3173396; doi:10.1371/journal.pone.0024368)

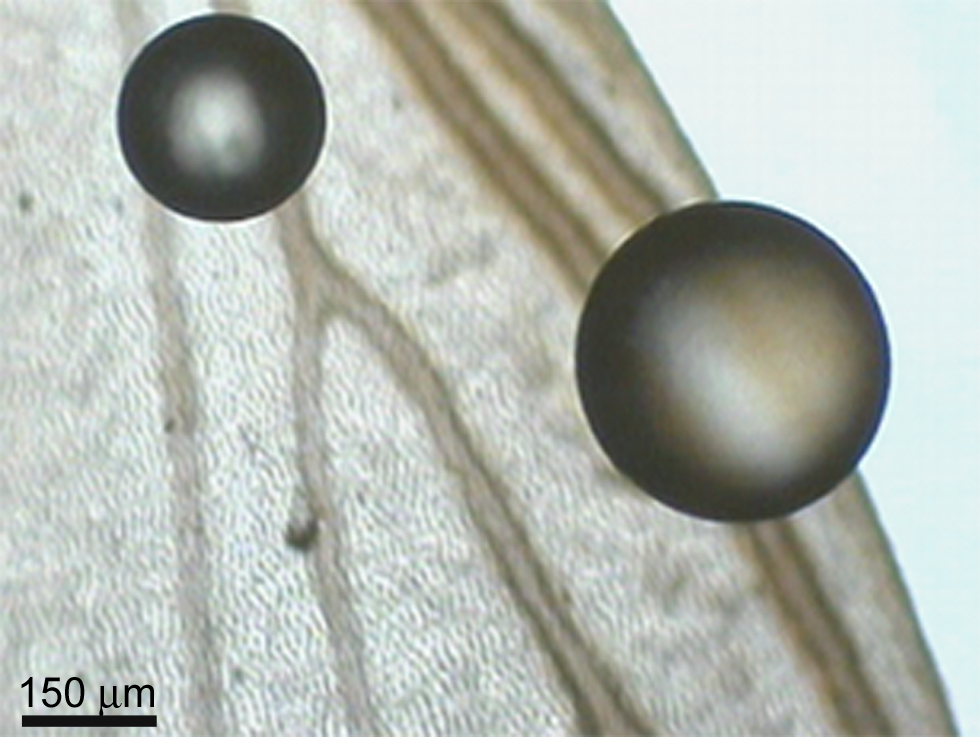

Supplement: Figure S1 — Optical microscope image showing large sized droplets resting on the wing surface of Nasutitermes sp. (TIF) [file pone.0024368.s001.tif]

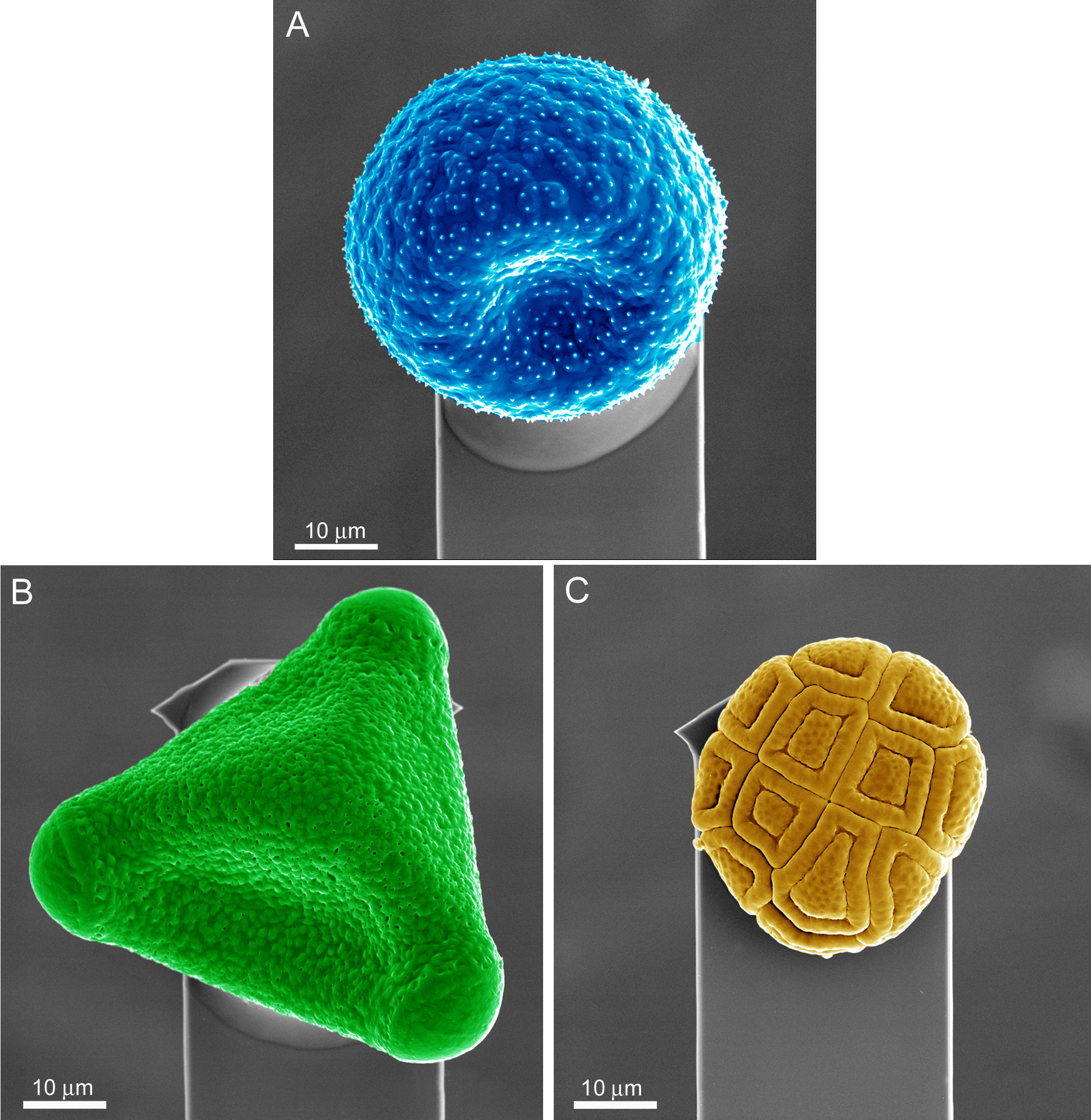

Supplement: Figure S2 — SEM images revealing the surface topographies of the three pollen grains ((A) Pimelea linifolia ssp. (B) Grevillea Red Sunset (C) Acacia fimbriata) attached to AFM tipless beam shaped levers. (TIF) [file pone.0024368.s002.tif]
